# Effects of Nebivolol on Endothelial Function and Exercise Parameters in Patients with Slow Coronary Flow

**DOI:** 10.4137/cmc.s3725

**Published:** 2009-11-03

**Authors:** Selma Tiryakioglu, Osman Tiryakioglu, Hasan Ari, Mehmet C. Basel, Tahsin Bozat

**Affiliations:** 1 Bursa Acibadem Hospital, Department of Cardiology, Bursa, Turkey.; 2 Bursa Yuksek İhtisas Training and Research Hospital, Department of Cardiovascular Surgery, Bursa, Turkey.

**Keywords:** coronary artery disease, nebivolol, brachial artery, vasodilation

## Abstract

**Objective:**

Earlier studies have reported that a decrease in exercise capacity might indicate endothelial dysfunction. However, the effects of improvement of endothelial functions on exercise capacity have not been evaluated. The aim of the present study is to investigate the effects of nebivolol on flow-mediated dilatation (FMD), and on the exercise capacities of the patients with slow coronary flow (SCF).

**Methods:**

The study population included 25 subjects with SCF (Group 1) documented by the thrombolysis in myocardial infarction (TIMI) frame count, and 25 control group (Group 2) subjects with normal coronary angiography, for a total of 50 subjects who underwent coronary angiography due to several indications and had no coronary lesion. The TIMI frame count (TFC) values of the subjects in Group I for left anterior descending artery, right coronary, and circumflex coronary artery were 61.8 ± 30.6, 37.2 ± 17.4, and 34.6 ± 17.4, respectively. All the subjects received nebivolol 5 mg/day.

**Results:**

At the end of the first month of FMD, the mean exercise duration (MED) and the Duke Scores of the patients with SCF were significantly higher than the baseline values. However, the values by the sixth month did not differ from that at the first month. Although a numerical improvement compared to the baseline values was observed for the subjects in Group 2 by the measurements at the end of the first and the sixth month, this difference was not statistically significant.

**Conclusions:**

Nebivolol treatment increases FMD in the subjects with SCF. The difference in the exercise parameters of these subjects is particularly dramatic, and such an outcome may indirectly indicate long-term improvement in endothelial function.

## Introduction

Slow coronary flow (SCF) phenomenon is a microvascular disease diagnosed by detection of slow passage of the contrast agent in the absence of epicardial occlusive disease. In 1972, Tambe et al first described the SCF phenomenon in six patients with chest pain.^1^ It is known that in patients detected with SCF, flow-mediated dilatation (FMD) is significantly impaired compared to the patients with normal coronary flow. Consequently, endothelial dysfunction plays an important role in the pathogenesis of SCF phenomenon.^2^ The benefit ratios of these patients using conventional antianginal drugs are quite low. While it has been reported that the decrease in exercise capacity might indicate endothelial dysfunction, the effects of improvement of endothelial functions on exercise capacity were not evaluated. It is known that endothelial dysfunction is present in the patients who are detected with SCF.^3^ Nebivolol (Vasoxen 5 mg tablets), a third-generation beta-blocker agent, may improve endothelial functions in these patients. Exhibiting the relationship between SCF progression and endothelial dysfunction is important.^4^ Therefore, nebivolol, an agent that improves endothelial dysfunction by inducing L-arginine/nitric oxide pathway-dependent vasodilatation, was used in the present study. The effects of nebivolol on FMD and exercise capacities of the patients with SCF were investigated.

## Methods

Patients who had undergone coronary angiography between October 2003 and May 2008 were screened, and 25 subjects with SCF detected by angiography were included in Group 1. The control group (Group 2) also included 25 subjects with normal coronary flow in the coronary angiography performed at the same period. The hospital Ethics Committee approved the study protocol, and each subject signed a written informed consent prior to enrolment. The demographic characteristics of the two groups were similar. No coronary lesion was present in any of the subjects.

SCF was defined as presence of thrombolysis in myocardial infarction (TIMI) Grade 2 flow in at least one of the coronary arteries angiographically with stenosis of less than 40% or without any lesion. The diagnosis was made by two independent and experienced cardiologists and a cardiovascular surgeon. Excluded from the study were subjects with TIMI Grade 2 flow after a coronary intervention, left ventricular ejection fraction of less than 50%, valvular insufficiency, conduction disorders of sinus and atrioventricular nodes, known peripheral vascular disease or diabetes mellitus, restrictive; hypertrophic or dilated cardiomyopathy, left ventricular hypertrophy; vasculitis and pulmonary renal or hematologic disease, and those who needed to receive additional medication.

The “TIMI frame count” method by Gibson et al was used to measure the opaque material and to detect the SCF pattern.^5^ The first frame was defined as the frame in which the opaque material entered coronary artery ostium and the coronary artery was reached, and the last frame was defined as the one needed for the imaging of the distal landmark by the opaque material. Distal landmarks were distal bifurcation for the left anterior descending artery (LAD), end of the distal bifurcation for the circumflex artery (Cx), and the first side branch of posterolateral artery (PLA) for the right coronary artery (RCA). The frame count of each artery was calculated by subtracting the first frame number from the last frame number. The TIMI frame count of LAD was significantly higher than the TIMI frame count of RCA and Cx, since the distance between the proximal and distal bifurcations of the LAD was longer than the other coronary arteries. Taking account of the requirement for the standardization of LAD frame (which is longer compared to other coronary arteries), Gibson attained a correction factor of 1.7 by dividing the TIMI frame count of LAD by the mean of combined frame count of Cx and RCA. The reference values (mean ± standard deviation) of the normal frame counts required for filling up coronary arteries, corrected according to the length of coronary artery, were 36 ± 1 for the LAD, 22.2 ± 4 for the Cx, and 20.4 ± 3 for the RCA. In the present study, in Standard 2 or above, mean values were used, while the values above 38 for the LAD, 30 for the Cx, and 26 for the RCA were accepted as SCF.^6^–^8^

Twenty-five subjects with documented SCF defined according to the TIMI frame count method included 13 females and 12 males with a mean age of 56.1 ± 7.9 years. The mean frame count of the subjects in this group was found to be 61.8 ± 30.6 for the LAD, 37.2 ± 17.4 for the RCA, and 34.6 ± 17.4 for the Cx.

The control group consisted of 10 males and 15 females. The mean age of the subjects was 52.1 ± 8.4 years. The mean frame count of the subjects in the control group was calculated to be 31.4 ± 11.3 for the LAD, 22.6 ± 5.7 for the RCA, and 20.3 ± 7.1 for the Cx (Table 1).

The measurement of FMD and the exercise test were carried out for each of the subjects. In all the subjects, endothelial function was assessed by measuring FMD using ultrasonographic examination of the forearm. All the patients were instructed to abstain from alcohol and caffeine 12 h before the test. The test was performed at appropriate room temperature after the subjects had fasted for 10–12 h. High resolution Doppler ultrasound (HDI-5000; ATL, Bothell, Washington) was used for the test. Following the measurement of base-line brachial artery diameter, a sphygmomanometer cuff was placed 5 cm above the last measurement location and inflated to 220 mmHg. The air in the cuff was subsequently released after 5 min. The sudden increase of the flow caused by the release of the cuff-induced FMD. FMD rates were determined 30s after the release of the cuff, and the third measurement was done after 15 min. Sublingual nitroglycerine was administered to evaluate nitrate-induced dilatation, and measurements were performed after 3–4 min. The brachial artery diameter was measured from the anterior to the posterior interface between the media and the adventitia, and determined at end-diastole. An average of three mean measurements from three cardiac cycles was used. Two independent researchers (a cardiologist and a radiologist) evaluated the measurements,^9^ Flow-mediated vasodilation was calculated as the percentage change in artery diameter from baseline to reactive hyperemia. Nitroglycerin-mediated vasodilation was also calculated by the same technique.

All the subjects in Group 1 and 2 received nebivolol 5 mg/day orally (Vasoxen 5 mg tb). FMD and the exercise test were repeated after one month and then six months later.

## Results

FMD values obtained one month after the initiation of treatment with nebivolol, were significantly higher than the baseline values in the group of patients with SCF (p < 0.001). The MED increased from 5.9 ± 2.1 to 8.3 ± 1.9, which was statistically significant (p = 0.016). The Duke Score was also evaluated as statistically higher (p < 0.001) (Table 2).

No significant difference in any of the three parameters was observed during evaluation of the values obtained at the first and six months, respectively (Table 2).

In terms of the baseline values of the control group (Group 2) and the study group (Group 1), the FMD was better in the control group (Group 2) (p < 0.001) (Table 3).

Although first month values of the subjects in Group 1 and Group 2 were higher compared to baseline values, it was observed that despite the increase, the FMD in Group 1 was still lower than that in Group 2 (p = 0.001) (Table 4).

It was observed that all the three parameters showed improvement in the sixth month. However, FMD in the study group was statistically lower compared with the control group (Table 5).

## Discussion

In earlier studies, it was reported that the decrease in exercise capacity might indicate endothelial dysfunction. However, the effects of the improvement of endothelial functions on exercise capacity were not evaluated.

Most patients with SCF have symptoms and signs of clinical coronary artery disease. About 4% of SCF patients are hospitalized due to unstable angina pectoris. An estimated 80% have recurrent anginal complaints, while 20% are again hospitalized due to angina pectoris.^1^,^3^,^6^

Although patients with normal coronary arteries who experience chest pain are generally thought to have good prognosis,^7^ it is believed that they are not entirely safe owing to the continued presence of symptoms,^8^ and possibly acute coronary syndrome risk.^2^ Przybojewski and Becker have reported that patients with SCF might experience myocardial infarction, but it is not known whether this has any effect on increasing thrombosis.^9^ Atak et al reported that QT dispersion associated with ventricular arthymia risk as well as cardiovascular mortality increased in patients with SCF.^10^

It is not known whether there will be an occurrence of thrombosis of the coronary artery among these patients in the future. However, fast coronary flow has several benefits, including improved quality of life and prevention of future complications.

Through the use of a non-invasive technique, we tried to assess whether the coronary flow increased or not. For this purpose, we used the brachial artery FMD technique, a test that is frequently used and commonly accepted.

Nebivolol is a novel, potent, and selective Beta-1 adrenergic receptor inducing endothelium-dependent arterial and venous vasodilatation via L-arginine/nitric oxide pathway. Nebivolol reduces the blood pressure and peripheral vascular resistance. Furthermore, it does not depress the functions of left ventricle in healthy subjects and hypertensive patients, and may even achieve improvements. In earlier studies, nebivolol did not only improve the distensibility and compliance of great arteries, but also reduced left ventricular hypertrophy in hypertensive patients.^4^

Nebivolol treatment does not effect, but sometimes may improve exercise tolerance. In hypertensive patients, one dose of nebivolol is effective for 24 h, with an absolute peak-trough ratio. The hypotensive effect of nebivolol is independent of age, weight, smoking habits, alcohol consumption, and diabetes. Nebivolol treatment is effective in both young and old populations, and its antianginal efficacy prevents hemodynamic deterioration in patients with stable congestive heart failure.^1^,^4^,^11^

The antiproliferative effects of nebivolol have been observed in endothelial and smooth muscle cell cultures. Infusion of nebivolol causes vasodilatation in all vascular beds as a result of endothelium-dependent mechanisms and the stimulation of Beta-3 adrenoreceptors,^11^ In addition, Kurtoğlu et al have shown that SCF could become normal with dipyridamole treatment.^12^

Due to the benefits mentioned earlier, nebivolol was used in the patient group for whom endothelial dysfunction was considered. It was found that the FMD parameters of brachial artery, and more importantly, the exercise parameters, improved significantly in patients with SCF.

In conclusion, nebivolol increases FMD in patients with SCF. The difference in the exercise parameters of these subjects is particularly dramatic, and such an outcome may indirectly indicate long-term improvement in endothelial function.

## Figures and Tables

**Table 1.** Demographic characteristics of the subjects.

	**Group 1 N** = **25**	**Group 2 N** = **25**	**P**
Age (years)	56.1 ± 7.9	52.1 ± 8.4	NS
Male	12 (48%)	10 (40%)	NS
Female	13 (52%)	15 (60%)	NS
Smoking	6 (24%)	5 (20%)	NS
COPD	1 (4%)	2 (8%)	NS
BMI (kg/m^2^)	27.1 ± 0.9	26.2 ± 1.0	NS
Systolic blood pressure (mmHg)	125 ± 5	126 ± 4	NS
Diastolic blood pressure (mmHg)	77 ± 3	76 ± 3	NS
Heart rate (beat/min)	67 ± 4	68 ± 5	NS
Hemoglobin (g/dL)	14.1 ± 1.2	14.3 ± 1.3	NS
LDL cholesterol (mg/dL)	121 ± 9	118 ± 7	NS
Frame count			
LAD	61.8 ± 30.6	31.4 ± 11.3	0.002
CX	34.6 ± 17.4	22.6 ± 5.7	0.044
RCA	37.2 ± 17.4	20.3 ± 7.1	0.001

**Abbreviations:** LAD, left anterior descending artery; CX, circumflex coronary artery; RCA, right coronary artery; BMI, body mass index. NS, not significant (p > 0.05).

**Table 2.** Values in the study group during the first month and six months after starting nebivolol treatment.

**Study Group**	**Baseline**	**First month**	**Sixth month**
FMD	0.0306 ± 0.01	0.0426 ± 0.01*	0.435 ± 0.01
MED	5.9 ± 2.1	8.3 ± 1.9**	8.1 ± 2.0
DUKE Score	1.3 ± 1.9	4.2 ± 1.6*	4.1 ± 1.7

**Abbreviations:** FMD, flow-mediated dilatation; MED, mean exercise duration.

**Notes:** *p < 0.001;

** p = 0.016.

**Table 3.** The difference between the groups before the study.

	**Group 1**	**Group 2**	**P**
FMD	0.0306 ± 0.01	0.0685 ± 0.02	<0.001
MED	5.9 ± 2.1	6.1 ± 2.2	NS
DUKE Score	1.3 ± 1.9	2.1 ± 2.3	NS

**Abbreviations:** FMD, flow-mediated dilatation; MED, mean exercise duration. NS, Not significant (p > 0.05).

**Table 4.** Comparison of the first month values of both groups.

**First month**	**Group 1**	**Group 2**	**P**
FMD	0.0426 ± 0.01	0.0672 ± 0.01	0.001
MED	8.3 ± 1.9	7.2 ± 1.7	NS
DUKE Score	4.2 ± 1.6	3.9 ± 1.5	NS

**Abbreviations:** FMD, flow-mediated dilatation; MED, mean exercise duration. NS, Not significant (p > 0.05).

**Table 5.** Comparison of the sixth month values of both groups.

**Sixth month**	**Group 1**	**Group 2**	**P**
FMD	0.435 ± 0.01	0.0691 ± 0.02	0.001
MED	8.1 ± 2.0	8.4 ± 2.1	NS
DUKE Score	4.1 ± 1.7	4.2 ± 1.6	NS

**Abbreviations:** FMD, flow-mediated dilatation; MED, mean exercise duration. NS, not significant (p > 0.05).
